# Proteomics of Skeletal Muscle: Focus on Insulin Resistance and Exercise Biology

**DOI:** 10.3390/proteomes4010006

**Published:** 2016-02-04

**Authors:** Atul S. Deshmukh

**Affiliations:** The Novo Nordisk Foundation Center for Protein Research, Faculty of Health and Medical Sciences, University of Copenhagen, Blegdamsvej 3b, 2200 Copenhagen, Denmark; atul.deshmukh@cpr.ku.dk; Tel.: +45-35-33-69-80

**Keywords:** mass spectrometry, diabetes, exercise adaptations, post-translational modifications, glucose, fat, secretome

## Abstract

Skeletal muscle is the largest tissue in the human body and plays an important role in locomotion and whole body metabolism. It accounts for ~80% of insulin stimulated glucose disposal. Skeletal muscle insulin resistance, a primary feature of Type 2 diabetes, is caused by a decreased ability of muscle to respond to circulating insulin. Physical exercise improves insulin sensitivity and whole body metabolism and remains one of the most promising interventions for the prevention of Type 2 diabetes. Insulin resistance and exercise adaptations in skeletal muscle might be a cause, or consequence, of altered protein expressions profiles and/or their posttranslational modifications (PTMs). Mass spectrometry (MS)-based proteomics offer enormous promise for investigating the molecular mechanisms underlying skeletal muscle insulin resistance and exercise-induced adaptation; however, skeletal muscle proteomics are challenging. This review describes the technical limitations of skeletal muscle proteomics as well as emerging developments in proteomics workflow with respect to samples preparation, liquid chromatography (LC), MS and computational analysis. These technologies have not yet been fully exploited in the field of skeletal muscle proteomics. Future studies that involve state-of-the-art proteomics technology will broaden our understanding of exercise-induced adaptations as well as molecular pathogenesis of insulin resistance. This could lead to the identification of new therapeutic targets.

## 1. Introduction

The prevalence of obesity and Type 2 diabetes is rising at an astronomical rate both in developed and developing countries. Increasing evidence links this rise to the population exercising less and becoming more sedentary, coupled with increased consumption of high caloric food. Type 2 diabetes is a progressive metabolic disorder caused by both genetic and environmental factors [1]. The pathogenesis of Type 2 diabetes involves functional defects in all major organs governing metabolic control including skeletal muscle, adipose tissue, and liver and pancreatic β-cells [1]. These defects lead to an impaired capacity of insulin to regulate whole body glucose homeostasis, a condition commonly known as “insulin resistance”. Impairments in insulin action in skeletal muscle have been clearly established as one of the early and primary defects in the pathogenesis of Type 2 diabetes [2,3,4]. This is not surprising as skeletal muscle is one of the largest tissues in human body and accounts for up to 80% of insulin-stimulated glucose uptake [5]. Therefore, the role of impaired insulin action on glucose metabolism in skeletal muscle should not be underestimated.

Like insulin, physical exercise has profound effects on glucose homeostasis. Regular physical activity can reduce the risk of developing Type 2 diabetes [6,7,8], while physical inactivity serves as a major risk factor for the development of insulin resistance and Type 2 diabetes [9]. The beneficial effects of exercise are partially mediated by extensive metabolic and molecular modeling of skeletal muscle [10]. Thus, together with the pathophysiology of insulin resistance in skeletal muscle, understanding the molecular regulation of exercise signaling and metabolism is crucial in guiding the development of future therapies to treat diabetes and/or advise health policies. Various “omics” approaches, including genomics, proteomics and metabolomics, are highly suited to undertake such investigations and might help to discover novel targets for prevention and/or treatment of Type 2 diabetes. Because the majority of the cellular processes are controlled by proteins, proteomics technology offers enormous promise for investigating molecular mechanisms underlying skeletal muscle insulin resistance and exercise-induced adaptation.

Liquid chromatography (LC) and high-resolution mass spectrometry (MS)-based proteomics have advanced tremendously over the years and currently have a profound impact in the field of biology and biomedicine [11]. They have also begun to advance molecular understanding of several muscle related diseases [12,13]. In order to apply the system biology approach and to investigate entire cellular system, it is desirable to monitor how all expressed proteins change under the process of interest. Recent technological advances now allow complete proteome of simple organisms like yeast [14] and near exhaustive proteomes of mammalian cells [15,16,17,18]. However, comprehensive proteomics of complex samples such as tissues in general and skeletal muscle in particular is challenging [19].

## 2. Skeletal Muscle Proteomics—Technical Challenges

### 2.1. Complexity of Skeletal Muscle Tissue

Skeletal muscle fibers are the most abundant cellular entities of the mammalian body. It represents 40% of the body mass in healthy human and plays vital role in locomotion, survival and whole body metabolism. These vital functions are mainly performed by contractile, and associated proteins, which accounts for >50% of total muscle mass [20]. This includes some of the giant proteins such as nebulin and titin with molecular masses of 800 kDa and 1200 kDa, respectively. The highly abundant contractile and associated proteins including myosin, troponin, tropomyosin, nebulin and associated proteins dramatically increases the dynamic range of the expressed proteome, which extends down to low-abundant proteins such as transcription factors [20]. The wide dynamic range coming from highly abundant proteins possesses one of the major problems in skeletal muscle proteomics (explained in Section 2.2).

Skeletal muscle fibers are highly plastic, meaning it can undergo considerable changes during physiological adaptations under exercise training, natural muscle ageing, and various pathological conditions such as insulin resistance, cachexia, and neuromuscular diseases [21,22,23]. These changes are associated with change in expression of protein or its specific isoforms and/or posttranslational modifications (PTMs). Based on the myosin heavy chain isoforms, skeletal muscle fibers are classified into slow oxidative, fast oxidative-glycolytic and fast glycolytic fibers as well as variety of hybrid muscle fiber [24]. An individual skeletal muscle consists of different amount of fiber types, hence possesses different metabolic properties. Fiber type ratio (determined by myosin heavy chain isoform) is constantly changing under physiological adaptations (e.g., exercise and ageing) and different pathological conditions (e.g., insulin resistance and cachexia) [24]. Histological studies have shown that the muscle fibers belonging to the same motor unit are metabolically similar or identical [25,26]. Therefore, it is likely that the metabolic properties of individual muscle fibers are primarily under neural control. The existence of spectrum of fibers makes skeletal muscle extremely heterogeneous, which is metabolically suited to a wide range of functional demands; however, the resulting diversity hampers proteomic analysis of skeletal muscle.

Human genome is relatively stable and comprises mere 20,000 protein coding genes [27]. Nevertheless, alternative splicing translates human genome into hundreds thousands of different protein species, extending proteomics complexity [28]. For instance, alternative splicing of skeletal muscle *SERCA* genes producing different isoforms Ca^2+^-ATPases and their PTMs leads to the formation of more than 10 different isoforms of *SERCA* [29,30]. Recently, it has been reported that human skeletal muscle consists of >23,000 transcripts [31]. Even though existence of protein isoforms provides the cell with a considerable degree of complexity, it is the ability of proteins and their isoforms to undergo PTMs that exponentially increases the protein diversity. Thus plasticity of muscular system together with its increased protein diversity due to alternate slicing and PTMs greatly impedes proteomic analysis of skeletal muscle.

**Figure 1:**
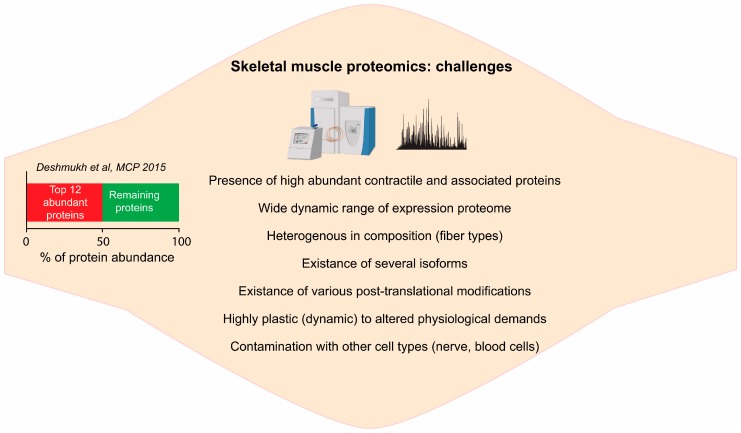
Challenges in skeletal muscle proteomics: summary of the various challenges in skeletal muscle proteomics.

The neuromuscular system is highly complex, consisting various fiber types, capillaries, satellite cells and several layers of connective tissues, with possible variations of their relative proportion under several pathophysiological conditions [24]. Skeletal muscle biopsies from rodents or human are highly heterogeneous and often contaminated with other cell types such as motor neurons and proteins originated from the blood. For instance, we have recently shown the presence of the proteins originated from nerves cells and blood cells in mouse muscle proteome [20]. Therefore one should take an account of protein abundance from mixed cell population when interpreting the results. The contamination of muscle cells by other cell types, to a certain degree, can be circumvented by studying pure single muscle fibers. We have recently shown that with the current technology, quantitative MS-based proteomics can be performed on single pure muscle fiber [32]. However, a tiny amount of protein obtained from single muscle fiber can be a limiting factor when performing PTMs studies or deep proteome studies where fractionation is required. In summary, wide dynamic range by highly abundant proteins, existence of different isoforms, PTMs, plasticity and heterogeneity of skeletal muscle poses huge challenges to proteomic analysis (Figure 1).

### 2.2. Deep Proteome of Skeletal Muscle Tissue

In the age of whole-genome analysis and system biology, the proteomics community is aiming to identify and quantify all expressed proteins in a given biological system (complete proteome). This is already possible for simple organism like yeast [14] but it is a colossal task for skeletal muscle tissue (described in Section 2.1). Skeletal muscle proteomics have already advanced molecular understanding of several muscle diseases but early studies had limited proteome coverage and lacked robust quantitation [23]. These studies often involved quantification of most abundant proteins such as contractile proteins and enzymes of metabolic pathways while the quantitation of low abundant regulatory proteins was missing. Deeper coverage of muscle proteome is indispensable for understanding the complex molecular events associated with exercise adaptation or insulin resistance (or any other pathological condition). Recently, using advance liquid chromatography coupled with mass spectrometry (LCMS) and streamlined bioinformatics analysis, we detected >8000 proteins including skeletal muscle transcription factors such as myod1, myogenin and other low abundant circadian clock proteins [20]. These low abundant transcriptional regulators were barely detected in previous proteomics studies. Contrary to skeletal muscle tissue proteome, proteome of C2C12 muscle cells is less challenging. In a similar study, we identified ~10,000 proteins in C2C12 cells [20]. Even though C2C12 myotubes is a commonly used model system in the field of muscle biology, they lack the 3D structure and specialized muscle functions characteristic of the tissue context. Therefore, it is desirable to perform the proteomics analysis of skeletal muscle tissue.

Our deep proteome analysis of skeletal muscle tissue revealed that the dynamic range of muscle proteome is spread over eight orders of magnitude. The top two most abundant proteins, myosin and titin, accounted for 18% and 16% of total protein mass, respectively, while the top 12 most abundant proteins already make up 50% of total protein mass [20] (Figure 1). When we ranked proteins according to their abundances, the lower half of the proteome accounted for negligible fraction of total protein mass (<0.1%). Proteins annotated with contractile machinery (Gene Ontology Cellular Compartment (GOCC)), the major contributors to increased dynamic range, constituted 53.6% of total proteins mass. Deep proteomic analysis also revealed detailed metabolic map of the skeletal muscle. More than 30% proteins were annotated to metabolic process (Gene Ontology Molecular Function (GOMF)), while 7% proteins to mitochondria (GOCC) and roughly 10% were annotated to enzymes of core metabolic pathways (glycolysis, krebs cycle, fatty acid oxidation and oxidative phosphorylation (OXPHOS)) [20]. Accurate quantitation of the enzymes of metabolic pathways is central to studying insulin resistance and exercise adaptations in skeletal muscle.

## 3. Emerging Technology for Skeletal Muscle Proteome

**Figure 2:**
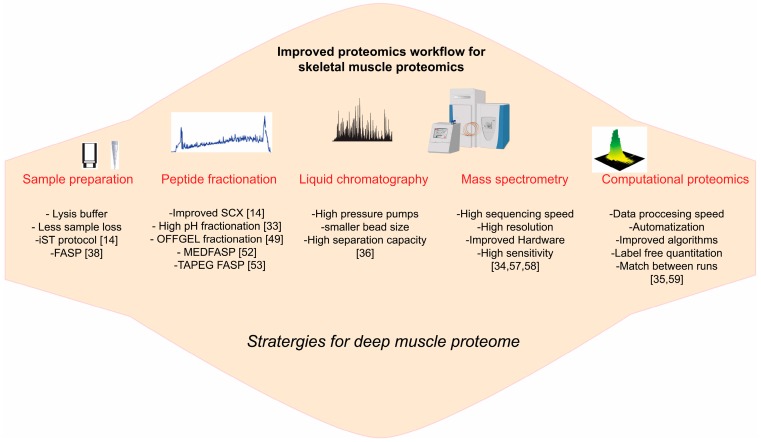
Skeletal muscle proteomics workflow: outline of the emerging development in the various preparative and analytical steps involved in the routine mass-spectrometry based proteomics. These advanced proteomics technologies have not yet been fully exploited in the field of skeletal muscle proteomics.

MS based proteomics is rapidly growing field with constant advent of varieties of sophisticated technologies with regards to sample preparation, peptide fractionation, LCMS instrumentation and bioinformatics analysis [14,33,34,35,36]. These technologies have not yet fully exploited in the field of skeletal muscle proteomics (Figure 2).

### 3.1. Sample Preparation

Sample preparation in proteomics workflow is an important step because it determines overall quality of proteomics analysis [37]. Efficient protein extraction and solubilization is crucial for proteomics analysis. Various reagents such as sodium dodecyl sulfate (SDS) [38], Na-deoxycholate [14], Na-laurate [39], or guanidine-hydrochloride [40] can be used for protein extraction. Recently, we tested these reagents for skeletal muscle protein extraction and found that SDS-based extraction provides highest protein extraction yields [41]. Even though SDS ensure complete lysis and solubilization of skeletal muscle, it need to be removed before subsequent enzymatic digestion using in-gel- [42], in-solution- [43] or protein reactor-based filters [38]. Indeed, in our deep muscle proteome analysis, we lysed the skeletal muscle tissue using SDS-based buffer and performed the enzymatic digestion on the reactor-based filters (FASP method) [20]. Urea-based lysis buffers are widely used for proteomics samples preparations. Nevertheless, Guanidine-hydrochloride and sodium deoxycholate have advantages over urea as the lysis agent because of their temperature stability and chemical inertness [40,44]. A recently published In-stage Tip (iST) [14] sample preparation protocol utilizes these lysis agent. The iST method is simple, scalable, robust, and highly sensitive and guarantees minimal sample loss [14]. This method has been tested for single muscle fiber proteomics [32], however, it needs to be tested for deep muscle proteome analysis.

### 3.2. Protein, Peptide Fractionation

With modern technology, single LC-MS/MS analysis (single-shot) using long columns (50 cm) and gradient coupled to a Orbitrap instrument can yield nearly complete proteome of single organisms like yeast [14,45]. However, LC-MS instruments have limitations with respect to single-shot analysis of highly complex protein mixtures particularly those who possess a wide dynamic range (such as skeletal muscle). Therefore, to reduce the sample complexity, additional separation steps such as fractionation at the levels of organelle, protein or peptide are favored.

Protein complexity can be reduced by protein fractionation prior to enzymatic digestions. This can be achieved using various chromatographic methods, including ion exchange [46], reversed phase [47], hydrophobic interaction [48], size exclusion or the more popular SDS polyacrylamide gel electrophoresis (SDS PAGE) separation [42]. Alternatively, peptide can be fractionated using several methods like OFFGEL fractionation [49], high pH fractionation [33], strong cation exchange chromatography [50] or strong anion exchange chromatography [51]. The wide range of available fractionation methods makes it challenging to choose the best-suited method for skeletal muscle proteome. To our knowledge, with respect to skeletal muscle proteomics, there has been no study where “fair” comparison between these methods has been done. We used OFFGEL fractionation method to achieve deep skeletal muscle proteome [20]. Recent reports showed that six-fraction of strong cation exchange chromatography (SCX) outperformed six-fraction of strong anion exchange chromatography (SAX) and just three peptide fractions using poly (styrenedivinylbenzene) reverse phase sulfonate (SDB-RPS) resulted in higher peptide number than the six-fraction SAX approach [14]. However, these fractionation methods need to be tested on skeletal muscle peptides.

Complexity of the protein samples can also be reduced by stepwise protein cleavage and fractionation in filter format. For instance, it was demonstrated that consecutive digestion of proteins with multiple proteases in the filter aided sample preparation (MEDFASP) format allows generation of two peptide fractions with an overlap of only 2%–5%. Moreover, it significantly increased the number of peptide and protein identifications [52]. In fact, we used this approach to quantify key metabolic pathways in slow and fast skeletal muscle [41]. Protein coverage using MEDFASP was further improved by inclusion of extra step, which involves enrichment of cysteine-containing peptides as a third fraction (TAPEG FASP) [53]. This extra fraction reduced the complexity of skeletal muscle proteome, increased peptide and protein identification and the sequence coverage of skeletal muscle proteome [53]. Another filter based stepwise digestion method exploited the abundance-dependent Michaelis–Menten kinetics of trypsin digestion to selectively digests and deplete abundant proteins [54,55]. This approach led to identification of low abundant proteins in yeast [54]; however, this elegant approach has not been tested on skeletal muscle lysate. All fractionation methods described here utilizes physicochemical properties of proteins/peptides and they might yield different results depending on complexity of the samples. For skeletal muscle proteomics, depending on the biological questions, one should compare fractionation methods side by side and choose the optimal one.

### 3.3. LCMS Instrumentations 

Modern chromatography and mass spectrometry setup is playing a pivotal role in characterizing deep proteome in relatively short period of time [11]. The current generation of high-pressure high-performance liquid chromatography pumps, together with very small bead particles as column material and comparatively long columns, has resulted in high peptide-separation capacity [36,56]. Present day mass spectrometers are extremely fast, very sensitive, have very high resolutions and a greater dynamic range [34,57,58]. This array of features makes it possible to achieve sub-ppm range mass accuracy, which is an obvious requirement for accurate peptide identification [59]. Together with improvements in sample preparation protocols and computational proteomics (discussed below), modern LCMS instrumentations are a game changer in the field of proteomics.

### 3.4. Computational Proteomics

Over the last decade, together with analytical proteomics workflow, there has been immense advancement in the field of computational proteomics. Earlier, proteomics data processing time for single project took several months and lots of steps were performed manually. Today, massive datasets can be processed in a completely automated manner in relatively short time, without compromising statistical accuracy [59]. The sophisticated algorithms have improved protein quantification and identifications [35]. For instance “label free quantification” using “MaxLFQ” algorithm is one such example where peptide intensities are directly compared between the samples given that the samples are measured under identical conditions and data were obtained using high resolution MS [35]. The MaxLFQ is completely integrated into the MaxQaunt [59] and can be activated by one additional click. For accurate quantifications, metabolic labeling (such as SILAC) and chemical labeling are still widely used [60,61,62]. This is primarily because of their accuracy and robustness but the care must be taken that the samples are handled in the same way. Chemical labeling can be applied to any proteomics samples; however, because the labels are introduced later in sample processing, some of the advantages in robustness are lost. Additionally, depending on the label used, it can also be expensive for large studies. It was shown that both SILAC and MaxLFQ generate similar ratio distributions [63], suggesting that quantification accuracies obtained by MaxLFQ are comparable to those obtained with SILAC. Moreover, similar to isotope-labeled standards, label free quantification is capable of absolute quantifications [64]. Indeed using raw peptide intensities, we have performed absolute quantification of key metabolic pathways in slow and white skeletal muscle [41].

In bottom-up proteomics, often peptides sequences are identified by MS/MS database search engine. Using some elegant computational tools, it is now possible to increase the peptide identification beyond those that have been sequenced. This can be achieved by transferring the peptide identifications from sequenced peptides to non-sequenced or unidentified peptides by matching their mass and retention time (“matchbetweenruns” feature in MaxQuant) [35]. A prerequisite for this is that MS runs should be performed under identical chromatographic condition (similar gradient, columns length, column beads, *etc.*) and the peptide masses should be accurately determined. The “matchbetweenruns” feature in MaxQuant aligns the retention times between the runs and—based on the accurately determined masses—efficiently transfers the peptide identifications from one LC run to other LC runs where the peptide was not sequenced. We found that this approach is particularly attractive when the identifications are matched from less complex cell line to more complex tissue of same origin. For instance, when we matched the protein identification from C2C12 muscle cell lines to mouse adult skeletal muscle, protein identifications were boosted by about 30% [20]. In this study, we also showed that most of the proteins identified skeletal muscle by the “match between runs” were partially or exclusively of low abundance and include interesting classes of skeletal muscle specific transcription factors, nuclear receptors and circadian clock proteins [20].

Thus, mass spectrometry-based proteomics is rapidly evolving. The new proteomics technology is not yet fully exploited in the field of skeletal muscle proteome. In the future, application of sophisticated proteomics workflow to complex tissues such as skeletal muscle may unravel underlying mechanisms for exercise-mediated adaptation in skeletal muscle and various pathological conditions such as insulin resistance and Type 2 diabetes.

## 4. Proteome Profiling of Diabetic Skeletal Muscle

Skeletal muscle insulin resistance is a hallmark feature of Type 2 diabetes [65,66]. Under diabetic conditions, skeletal muscle undergoes significant remodeling, which results in decreased oxidative capacity, fiber type changes, contractile weakness, and insulin resistance [67]. Majority of these changes are attributed to abnormal changes in signaling events, protein expression and/or PTMs [22]. Therefore detailed proteomics and PTMs analysis of diabetic skeletal muscle can provide underlying causes for Type 2 diabetes.

Over the last decades, several research groups have performed skeletal muscle proteome analysis from diabetic patients and rodent models of diabetes [68,69,70,71]. These pioneering studies have already begun to advance our understanding of skeletal muscle insulin resistance. For instance, using proteomics and transcriptomics approach, Stentz and Kitabchi showed that the expression of key proteins (and genes) regulating glucose transport and glycolysis are differentially regulated in diabetic skeletal muscle compared to their healthy controls [70]. Another study that involved proteome analysis of lean, obese and Type 2 diabetic skeletal muscles revealed that the insulin-resistance muscle bears reduced expression of mitochondrial and structural proteins [72]. These abnormalities were also observed in skeletal muscle from rodent model of Type 2 diabetes [71]. Using two-dimensional electrophoresis combined with mass spectrometry, Choi *et al.* demonstrated gender specific differences in rodent model for Type 2 diabetes [73]. Altered proteins expression was also observed in human primary skeletal muscle cells from Type 2 diabetic subjects compared to their healthy controls. It was proposed that the derangements in proteasome system from Type 2 diabetes might have led to development of insulin resistance [74]. Proteomics screens of skeletal muscle can also reflect the metabolic state under disease conditions. For example, Giebelstein *et al.* showed that the abundance of glycolytic enzymes was up-regulated, while mitochondrial proteins were down-regulated in skeletal muscle from insulin-resistant subjects. Interestingly, these changes were associated with shift in muscle properties towards a fast-twitch pattern [68]. Numerous proteomics studies suggest pivotal role for mitochondrial function in development of Type 2 diabetes. A more recent study showed that skeletal muscle from rodent model for diabetes bear increased mitochondrial protein degradation and decreased protein synthesis, resulting in reduced abundance of proteins involved in mitochondrial respiration and beta oxidation [69]. These proteomics screens have documented hundreds of differentially regulated proteins in diabetic skeletal muscle. Functional relevance of these differently regulated proteins remains to be elucidated.

Due to technical challenges, majority of these studies had limited proteome coverage and lacked robust quantifications. With cutting age proteomics technology, it is now possible to achieve comprehensive proteome coverage of skeletal muscle [20]. Future studies using the state-of-the-art proteomics will broaden our understanding of Type 2 diabetes.

### 4.1. Diabetes and Skeletal Muscle PTMs

In skeletal muscle, binding of insulin to insulin receptor (IR) initiate a signaling cascade that results in translocation of the insulin-sensitive glucose transporter 4 (GLUT4) to plasma membrane [22]. This is the most important step in insulin stimulated glucose uptake. Studies from cellular, animal and human model for Type 2 diabetes have revealed clear impairment in insulin signaling cascade towards GLUT4 translocation. These impairments were primary documented using phospho-specific antibodies against few known insulin signaling molecules such as insulin receptor substrate (IRS), protein kinase B (PKB) and Rab GTPase activating proteins TBC1D4 [22]. It is likely that insulin signaling to glucose transport is not limited these known signaling events. Moreover, the function of these signaling molecules might be regulated by their expression levels and/or their PTMs. Earlier studies involving phosphoproteomics of healthy skeletal muscle (non-stimulated) revealed several phosphorylation proteins that are involved in regulation of mitochondrial metabolism, sarcomeric functions, muscle contraction, glucose and glycogen metabolism [75,76]. These pioneering studies significantly advanced our understanding of skeletal muscle biology but these studies had limited phosphoproteome coverage. MS-based proteomics has begun to reveal the true extent of the phosphorylation and acetylation landscape in various tissues and cells [33,77,78,79]. Large-scale phosphoproteomics studies have already deciphered hundreds of novel insulin regulated phosphoproteins in insulin sensitive tissues like liver and skeletal muscle cells [77,80]. To my knowledge, there has been no study comparing comprehensive phosphoproteome of control and diabetic skeletal muscle. In addition to phosphorylation and acetylation, other PTMs such as ubiquitination, glycosylation, formylation are also important for regulation of cellular process [81]. Current PTMs studies focus on phosphorylation and acetylation but other modifications are also becoming amenable for investigations. Future PTMs studies comparing control and diabetic skeletal muscle may help in understanding molecular pathogenesis of insulin resistance in skeletal muscle.

### 4.2. Diabetes, Skeletal Muscle Metabolism and Muscle Fiber Type

Type 2 diabetes patients are characterized by a decreases oxidative capacity and high circulating free fatty acids (FFA) [82]. Increased levels of plasma FFA are linked to the skeletal muscle insulin resistance [83]. Additionally, Type 2 diabetes is associated with impaired metabolic flexibility, *i.e.*, inability to switch from fatty acid to glucose oxidation in response to insulin [84]. Mechanism underlying these derangements in diabetic skeletal muscle remains elusive. Several studies claimed that the impaired mitochondrial function is responsible for development of Type 2 diabetes [85,86]. Whether muscular mitochondrial aberrations are cause or consequence of Type 2 diabetes is not clear. Nevertheless, these reports suggest that the way muscle handles fat and glucose is crucial to maintaining metabolic homeostasis. In fact, proteins controlling energy metabolism are the second most abundant protein category (after contractile proteins) in skeletal muscle [20], suggesting their importance in skeletal muscle metabolism. Since most of the enzymes of core metabolic pathways (glycolysis, beta oxidation, Krebs cycle, OXPHOS, and pentose phosphate pathways) are highly abundant in skeletal muscle, they can be easily identified and quantified MS [20,41]. For instance, we recently reported the metabolic map of slow oxidative and fast glycolytic skeletal muscle from mouse [41]. Compared to fast muscle, slow muscle has higher mitochondrial proteins content, higher concentrations of enzymes of beta oxidation, TCA cycle, OXPHOS and lower concentrations of glycolytic enzymes [41]. As expected, myosin heavy chain isoforms Myh 1,2,7 were higher in red muscle, while Myh 4 isoform was predominant in fast muscle [41]. Due to inherent properties of individual fibers, fiber type changes are always associated with change in their metabolic properties. Such analysis can be used to monitor fiber type transition in diabetic skeletal muscle. It has been reported that the skeletal muscle from Type 2 diabetic patients undergo fiber type transition from slow-to-fast fibers, which is associated with reduced activity of oxidative enzymes [87]. Proteomic profiling of metabolic enzymes, together with their PTMs, can provide more mechanistic understanding of insulin resistance in skeletal muscle.

### 4.3. Diabetes and Muscle Strength

Impaired structure and function of the contractile fiber (sarcopenia) are responsible for the fragility of sedentary elderly patients [88]. Patients with Type 2 diabetes showed greater decline in muscle mass and muscle strength with age [89], which may be a cause or a consequence of an altered proteome profile. In the same study, it was reported that the size of the fast muscle fibers was smaller in Type 2 diabetic muscles [89]. Few other studies showed clear link between decreased muscle strength and Type 2 diabetes [90,91]. Muscle mass is often measured by body scanning technique such as dual-energy x-ray absorptiometry (DXA), while muscle strength is estimated by various performance tests [89,90,91]. Contractile proteins, which constitutes >50% muscle proteome, are the most abundant protein category in skeletal muscle [20]. Whether muscle weakness is linked to decreased abundance and/or altered PTMs of contractile protein is still unknown. Future studies co-relating MS-based quantitation of contractile proteins with body mass and muscle strength will provide valuable information for reduced muscle strength in diabetic patients.

### 4.4. Skeletal Muscle Biomarkers for Diabetes

Type 2 diabetes is often underdiagnosed. About one-third of people with diabetes do not know they have it. The average lag between onset of Type 2 diabetes and the diagnosis is seven years, and that onset of Type 2 diabetes probably occurs at least 12 year before its clinical diagnosis [92]. Recently, it has been shown that the early detection and treatment of Type 2 diabetes reduces cardiovascular disease related morbidity and mortality [93]. Traditionally, Type 2 diabetes or prediabetes is diagnosed using only fasting glucose or glucose two hours during oral glucose tolerance test. Recently, plasma levels of glycated hemoglobin (HbA1c) are also used for diagnosis of Type 2 diabetes. All existing diagnostic methods have their advantages and disadvantages [94]. There is an absolute need to discover new biomarkers that can be used for early diagnosis and disease monitoring. Skeletal muscle proteomics promises to play a major role in the establishment of Type 2 diabetic specific biomarker signature. Such biomarkers signature can be crucial for the development of improved diagnosis, the monitoring of disease progression, assessment of drug action and the identification of novel therapeutic targets.

### 4.5. Interaction Proteomics

Interaction of proteins with other proteins, DNA, RNA, or metabolites, regulates numerous cellular and molecular functions in the cells. The size of the human interactome appears to be far more complex than the genome or proteome [95,96]. MS-based proteomics has had a significant impact on studying protein–protein interactions [96,97,98,99]. It has also started to unravel novel abnormalities along insulin signaling in skeletal muscle. For instance, interactome of Insulin receptor substrate 1 (IRS1) showed increased interaction of multiple proteins in skeletal muscles from obese and Type 2 diabetic subjects compared to their controls [100]. Future interactome studies of other signaling molecules along the canonical insulin signaling pathways might improve our understanding of insulin signaling and insulin resistance in skeletal muscle.

## 5. Proteomics Application to Study Exercise Biology

Physical inactivity (sedentary lifestyle) serves as a major risk factor for development of insulin resistance and Type 2 diabetes [9]. It is associated with decreased insulin sensitivity, attenuation of postprandial lipid metabolism, loss of muscle mass and accumulation of visceral adipose tissue [101,102]. Like insulin, exercise/muscle contraction is a major stimulator of skeletal muscle glucose uptake. A single bout of exercise or exercise training increases skeletal muscle glucose uptake in an insulin-dependent and insulin-independent manner [103,104,105]. Unlike insulin, exercise-stimulated glucose uptake is unaltered in skeletal muscle from insulin resistant humans or rodents, providing evidence that exercise-mediated signal transduction pathways are intact in diabetic muscle [106,107]. It is known that the acute exercise makes skeletal muscle more sensitive to insulin while lifestyle modification though regular (chronic) exercise reduces the incidence of subsequent diabetes by 60% [9,104,105]. Thus, exercise appears to play essential role in metabolic homeostasis and remains one of the most promising interventions for treatment of diabetes and obesity as well as the associated disorders. Therefore thorough investigation of MS-based protein profiles between control and exercised skeletal muscle may identify novel proteins with potential anti-diabetic effects. To better understand exercise effect on health, it is crucial to understand acute and long-term (chronic) effects of exercise on signaling cascade, metabolism and long term adaptation. The Section 5.1 and Section 5.2 describes how MS-based proteomics can be applied to the field of exercise biology.

### 5.1. Acute Exercise (Muscle Contraction) and PTMs

The effect of acute exercise on whole-body insulin sensitivity can be explained by exercise-induced signaling networks. In the past, majority of studies investigating exercise-induced signaling pathways were performed using immunoblotting techniques and phospho-specific antibodies against specific kinases. This led to the identification of several exercise-responsive kinases such as AMPK, PKA, CaMK, MAPK, PKC, FAK and mTOR [10,21,108]. However, it is likely that the exercise-mediated signaling is not limited to these kinases and their phosphorylation. In fact, MS-based phosphoproteomics studies have begun to unravel the complexity of exercise-induced protein phosphorylation. For instance, global phosphoproteome analysis of human skeletal muscle after high-intensity exercise bout revealed >1000 exercise-regulated phosphosites on 562 proteins [109]. Effects of exercise on other PTMs are relatively unexplored. McGee *et al.* showed for the first time that an acute bout of exercise led to increase in acetylation of histone 3 lysine 36 acetylation [110]. The acetylation of this conserved residue has been shown to be associated with transcriptional elongation. The existence of various PTMs and their possible interplay makes muscle exercise signaling landscape far greater than previously appreciated. In the future, large-scale proteomics studies investigating exercise-induced PTMs, their interplay and their relevance to whole body insulin sensitivity will unravel molecular basis for exercised mediated anti-diabetic effects.

### 5.2. Exercise Training and Skeletal Muscle Adaptations

Increased physical activity remains the primary preventive approach for metabolic diseases. In fact, regular physical activity combined with dietary intervention is more successful than pharmacological intervention in the treatment and prevention of Type 2 diabetes [111]. Skeletal muscle demonstrates remarkable malleability in functional adaptation in response to contractile activity. Repeated muscle contractions associated with the frequent exercise training are the potent stimuli for physiological adaptations [112]. Exercise training orchestrates numerous morphological and metabolic adaptations in skeletal muscle. This includes changes in contractile protein and function [113,114], mitochondrial function [115], metabolic regulation [116], intracellular signaling [117], and transcriptional responses [118]. Collectively, these changes lead to increased sensitivity to insulin enhanced capacity to oxidize glucose and fat and, despite well-established phenotypic changes, the molecular mechanisms underlying exercise-mediated skeletal muscle are poorly characterized. It is widely accepted that the exercise training induced adaptations are associated with alteration in protein content and enzyme activities. Several large-scale proteomics studies of human or rodent skeletal muscle have significantly improved our understanding of the exercise biology. Holloway *et al.* were the first to investigate the effects of exercise on human skeletal muscle proteome [119]. Using 2D gel analysis, they discovered 256 spots, of which 20 proteins were differentially expressed after six weeks of interval training. Training induced adaptations were associated with increased expression of mitochondrial proteins [119]. Another study involving 2D fluorescence difference gel electrophoresis (2DDIGE)-based analysis of human skeletal muscle proteome showed that the extensive remodeling of the mitochondrial proteome occurred after only seven days of exercise training [120]. Using FASP-based digestion method and OFFGEL fractionation, 3481 proteins were identified in human skeletal muscle; however, only 702 proteins could be identified in all samples [121]. Despite this, proteomics analysis of skeletal muscle from healthy endurance exercise-trained and untrained individuals showed clear differences in proteome profiles. Proteins associated with oxidative phosphorylation, tricarboxylic acid and fiber types were significantly up-regulated in trained individuals as opposed to untrained individuals [121]. Using MS-based proteomic analysis of skeletal muscle from sedentary and active mice, Alves *et al.* showed that sedentary mice presented significant loss of electron transport chain (ETC) functionality in opposition to active mice [122]. MS-based proteomics has also been performed in skeletal muscle from physically inactive individuals. In one such study, proteins involved in aerobic metabolism were significantly down-regulated in skeletal muscle from physically inactive subjects [123]. Very few studies using MS-based strategies have been published focusing on cross-talk between exercise training and pathophysiological conditions such as diabetes. In one such study, exercise training significantly altered the abundance of 17 proteins in skeletal muscle from Type 2 diabetes. These proteins were related to energy metabolism, the cytoskeleton, or few with unknown function [124]. Another study using diet-induced insulin resistant mice showed that six weeks of exercise training led to increased expression of 23 different proteins in skeletal muscle from exercised mice as compared to their sedentary controls. These proteins were mainly involved in antioxidative stress response, lipid binding, myofibrillar contraction, mitochondrial functions and molecular chaperons [125]. However, like any other skeletal proteomics studies, these pioneering studies had limited proteome coverage and lacked robust quantitation. Moreover, the role of various PTMs such as phosphorylation and acetylation in exercised-induced adaptations is not yet explored. Future studies involving modern proteomics technology will gain an understanding of the important role physical exercise plays in maintaining health.

## 6. Secretome of Insulin Resistant and Exercised Skeletal Muscle

**Figure 3:**
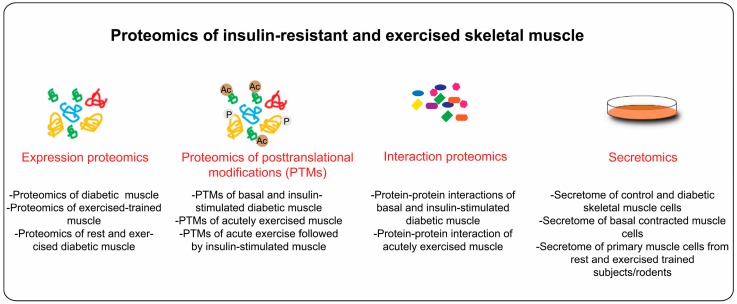
Proteomics of insulin-resistant and exercised skeletal muscle: the proteomics application of insulin resistant and exercised skeletal muscle can be broadly classified under three categories, expression proteomics (global protein expression profile), PTMs (posttranslational modifications), and secretomics (secretion profiles of muscle cells).

Over the last decade, skeletal muscle has emerged as an important secretory organ. Proteins or peptides secreted from skeletal muscle (often termed as myokines) can have autocrine, paracrine, and endocrine effects, which might influence whole body metabolism [126]. Therefore, proteomic analysis of secreted proteins from skeletal muscle holds enormous promise. This will particularly help us to understand how muscle communicates with other organs such as adipose tissue, brain and liver. Secretome analysis is often performed using serum free media from primary cell cultures or cell lines. As opposed to adult skeletal muscle proteomics, secretome analysis of skeletal muscle cells is relatively easy; however, it faces few other challenges, such as detection of *bona fide* secreted proteins at low concentration by MS (pg/mL) and separation of authentic secreted proteins from proteins derived from cell leakage or serum. With modern technology, quantitative MS-based secretome analysis of cells can be performed with pictogram sensitivity [127]. Using state-of-the-art MS and streamlined bioinformatics workflow, we recently showed that C2C12 muscle cells secretes >1000 high confidence secreted proteins [128]. Interestingly, 80% of these proteins are also found in adult skeletal muscle [128]. An attractive element of skeletal muscle cells is that they can be manipulated to mimic some aspects of skeletal muscle insulin resistance or muscle contractions *in vivo*. For instance, skeletal muscle insulin resistance can be achieved by treatment of muscle cells with high nutrients (amino acids, glucose, and lipids) [129,130,131] or pro-inflammatory factors such as tumor necrosis factor alpha (TNFα) [132]. Exercise/contraction-inducible responses in skeletal muscle can be studied using Electric Pulse Stimulation (EPS) of differentiated muscle cells (myotubes) [133]. These models have already been used for investigation of secretome of insulin resistant and exercised muscle. We recently showed that ~40% secreted proteins were regulated under lipid-induced insulin resistance conditions [128]. While using EPS stimulated human primary muscle cells, Raschke *et al.* identified and validated several novel contraction-regulated myokines [134]. Raschke *et al.* [134] used cytokines antibody arrays but similar analysis can be performed using MS-based proteomics. Thus, secretome analysis of insulin resistance and EPS stimulated muscle cells has begun to unravel the world of skeletal muscle secreted proteins. The function and regulation of newly identified secreted proteins in the context of muscle physiology are largely unexplored. Therefore, further studies are required to clarify their regulation, their roles in distinct signaling pathways and skeletal muscle metabolism. Figure 3 summarizes different proteomics approaches that can be applied for study of skeletal muscle insulin resistance and exercise-induced adaptations.

## 7. Conclusions

MS-based proteomics analysis of skeletal muscle is challenging. This is primarily due to the wide, dynamic range of highly abundant contractile and associated proteins; the heterogeneity; and various PTMs. High-resolution mass spectrometry-based proteomics has progressed tremendously over the years. Improved proteomics workflow at the level of sample preparation, liquid chromatography, mass spectrometry and computational analysis has enabled probing skeletal muscle proteome at an unprecedented depth [20]. These advanced proteomics technologies have not yet been fully exploited in the field of skeletal muscle proteomics.

Skeletal muscle is the largest depot for glucose storage in the body [5]. Insulin and exercise are the major stimulators of skeletal muscle glucose uptake. Skeletal muscle insulin resistance, as evident by impaired glucose uptake and lipid oxidation, is the primary defect in development of Type 2 diabetes [1,65]. It is known that a single bout of exercise makes skeletal muscle more sensitive to insulin, while lifestyle modification through regular exercise reduces the incidence of diabetes by 60% [9,104,105,111]. Both insulin resistance and exercise adaptations involve a complex metabolic process that defies explanation by a single protein or etiological pathway. Therefore, MS-based proteomics serves as an attractive tool for monitoring global proteome and PTMs changes in insulin resistance and exercised skeletal muscle. It has already begun to catalogue the diabetes or exercise regulated proteins and PTMs in skeletal muscle. Future studies involving state-of-the-art proteomics will broaden our understanding of exercise induced adaptation and molecular pathogenesis of skeletal muscle insulin resistance. Information generated from the proteome screens may one day be used by health care practitioners and exercise physiologist to identify people at risk for metabolic diseases and may help them design precision interventions to achieve maximal health benefits.

## References

[1] Kahn C.R. (1994). Banting Lecture. Insulin action, diabetogenes, and the cause of type II diabetes. Diabetes.

[2] Eriksson J., Koranyi L., Bourey R., Schalin-Jantti C., Widen E., Mueckler M., Permutt A.M., Groop L.C. (1992). Insulin resistance in Type 2 (non-insulin-dependent) diabetic patients and their relatives is not associated with a defect in the expression of the insulin-responsive glucose transporter (GLUT-4) gene in human skeletal muscle. Diabetologia.

[3] Henriksen J.E., Alford F., Handberg A., Vaag A., Ward G.M., Kalfas A., Beck-Nielsen H. (1994). Increased glucose effectiveness in normoglycemic but insulin-resistant relatives of patients with non-insulin-dependent diabetes mellitus. A novel compensatory mechanism. J. Clin. Investig..

[4] Vaag A., Henriksen J.E., Beck-Nielsen H. (1992). Decreased insulin activation of glycogen synthase in skeletal muscles in young nonobese Caucasian first-degree relatives of patients with non-insulin-dependent diabetes mellitus. J. Clin. Investig..

[5] Ferrannini E., Bjorkman O., Reichard G.A., Pilo A., Olsson M., Wahren J., DeFronzo R.A. (1985). The disposal of an oral glucose load in healthy subjects. A quantitative study. Diabetes.

[6] Helmrich S.P., Ragland D.R., Leung R.W., Paffenbarger R.S. (1991). Physical activity and reduced occurrence of non-insulin-dependent diabetes mellitus. N. Engl. J. Med..

[7] Manson J.E., Nathan D.M., Krolewski A.S., Stampfer M.J., Willett W.C., Hennekens C.H. (1992). A prospective study of exercise and incidence of diabetes among US male physicians. JAMA.

[8] Manson J.E., Rimm E.B., Stampfer M.J., Colditz G.A., Willett W.C., Krolewski A.S., Rosner B., Hennekens C.H., Speizer F.E. (1991). Physical activity and incidence of non-insulin-dependent diabetes mellitus in women. Lancet.

[9] Tuomilehto J., Lindstrom J., Eriksson J.G., Valle T.T., Hamalainen H., Ilanne-Parikka P., Keinanen-Kiukaanniemi S., Laakso M., Louheranta A., Rastas M. (2001). Finnish Diabetes Prevention Study G. Prevention of Type 2 diabetes mellitus by changes in lifestyle among subjects with impaired glucose tolerance. N. Engl. J. Med..

[10] Goodyear L.J., Kahn B.B. (1998). Exercise, glucose transport, and insulin sensitivity. Annu. Rev. Med..

[11] Mann M., Kulak N.A., Nagaraj N., Cox J. (2013). The coming age of complete, accurate, and ubiquitous proteomes. Mol. Cell.

[12] Doran P., Martin G., Dowling P., Jockusch H., Ohlendieck K. (2006). Proteome analysis of the dystrophin-deficient MDX diaphragm reveals a drastic increase in the heat shock protein cvHSP. Proteomics.

[13] Ohlendieck K. (2010). Proteomics of skeletal muscle differentiation, neuromuscular disorders and fiber aging. Expert Rev. Proteom..

[14] Kulak N.A., Pichler G., Paron I., Nagaraj N., Mann M. (2014). Minimal, encapsulated proteomic-sample processing applied to copy-number estimation in eukaryotic cells. Nat. Methods.

[15] Beck M., Schmidt A., Malmstroem J., Claassen M., Ori A., Szymborska A., Herzog F., Rinner O., Ellenberg J., Aebersold R. (2011). The quantitative proteome of a human cell line. Mol. Syst. Biol..

[16] Nagaraj N., Wisniewski J.R., Geiger T., Cox J., Kircher M., Kelso J., Paabo S., Mann M. (2011). Deep proteome and transcriptome mapping of a human cancer cell line. Mol. Syst. Biol..

[17] Wilhelm M., Schlegl J., Hahne H., Moghaddas Gholami A., Lieberenz M., Savitski M.M., Ziegler E., Butzmann L., Gessulat S., Marx H. (2014). Mass-spectrometry-based draft of the human proteome. Nature.

[18] Kim M.S., Pinto S.M., Getnet D., Nirujogi R.S., Manda S.S., Chaerkady R., Madugundu A.K., Kelkar D.S., Isserlin R., Jain S. (2014). A draft map of the human proteome. Nature.

[19] Ohlendieck K. (2011). Skeletal muscle proteomics: Current approaches, technical challenges and emerging techniques. Skelet. Muscle.

[20] Deshmukh A.S., Murgia M., Nagaraj N., Treebak J.T., Cox J., Mann M. (2015). Deep proteomics of mouse skeletal muscle enables quantitation of protein isoforms, metabolic pathways, and transcription factors. Mol. Cell. Proteom..

[21] Egan B., Zierath J.R. (2013). Exercise metabolism and the molecular regulation of skeletal muscle adaptation. Cell Metab..

[22] Deshmukh A.S. (2015). Insulin-stimulated glucose uptake in healthy and insulin-resistant skeletal muscle. Horm. Mol. Biol. Clin. Investig..

[23] Ohlendieck K. (2013). Proteomic identification of biomarkers of skeletal muscle disorders. Biomark. Med..

[24] Schiaffino S., Reggiani C. (2011). Fiber types in mammalian skeletal muscles. Physiol. Rev..

[25] Kugelberg E., Edstrom L. (1968). Differential histochemical effects of muscle contractions on phosphorylase and glycogen in various types of fibres: Relation to fatigue. J. Neurol. Neurosurg. Psychiatry.

[26] Vetter C., Reichmann H., Pette D. (1984). Microphotometric determination of enzyme activities in type-grouped fibres of reinnervated rat muscle. Histochemistry.

[27] Clamp M., Fry B., Kamal M., Xie X., Cuff J., Lin M.F., Kellis M., Lindblad-Toh K., Lander E.S. (2007). Distinguishing protein-coding and noncoding genes in the human genome. Proc. Natl. Acad. Sci. USA.

[28] Black D.L. (2003). Mechanisms of alternative pre-messenger RNA splicing. Annu. Rev. Biochem..

[29] Brini M., Carafoli E. (2009). Calcium pumps in health and disease. Physiol. Rev..

[30] Periasamy M., Kalyanasundaram A. (2007). SERCA pump isoforms: Their role in calcium transport and disease. Muscle Nerve.

[31] Lindholm M.E., Huss M., Solnestam B.W., Kjellqvist S., Lundeberg J., Sundberg C.J. (2014). The human skeletal muscle transcriptome: Sex differences, alternative splicing, and tissue homogeneity assessed with RNA sequencing. FASEB J..

[32] Murgia M., Nagaraj N., Deshmukh A.S., Zeiler M., Cancellara P., Moretti I., Reggiani C., Schiaffino S., Mann M. (2015). Single muscle fiber proteomics reveals unexpected mitochondrial specialization. EMBO Rep..

[33] Batth T.S., Francavilla C., Olsen J.V. (2014). Off-line high-pH reversed-phase fractionation for in-depth phosphoproteomics. J. Proteome Res..

[34] Scheltema R.A., Hauschild J.P., Lange O., Hornburg D., Denisov E., Damoc E., Kuehn A., Makarov A., Mann M. (2014). The Q Exactive HF, a Benchtop mass spectrometer with a pre-filter, high-performance quadrupole and an ultra-high-field Orbitrap analyzer. Mol. Cell. Proteom..

[35] Cox J., Hein M.Y., Luber C.A., Paron I., Nagaraj N., Mann M. (2014). MaxLFQ allows accurate proteome-wide label-free quantification by delayed normalization and maximal peptide ratio extraction. Mol. Cell. Proteom..

[36] Thakur S.S., Geiger T., Chatterjee B., Bandilla P., Frohlich F., Cox J., Mann M. (2011). Deep and highly sensitive proteome coverage by LC-MS/MS without prefractionation. Mol. Cell. Proteom..

[37] Altelaar A.F., Heck A.J. (2012). Trends in ultrasensitive proteomics. Curr. Opin. Chem. Biol..

[38] Wisniewski J.R., Zougman A., Nagaraj N., Mann M. (2009). Universal sample preparation method for proteome analysis. Nat. Methods.

[39] Lin Y., Huo L., Liu Z., Li J., Liu Y., He Q., Wang X., Liang S. (2013). Sodium laurate, a novel protease- and mass spectrometry-compatible detergent for mass spectrometry-based membrane proteomics. PLoS ONE.

[40] Poulsen J.W., Madsen C.T., Young C., Poulsen F.M., Nielsen M.L. (2013). Using guanidine-hydrochloride for fast and efficient protein digestion and single-step affinity-purification mass spectrometry. J. Proteome Res..

[41] Rakus D., Gizak A., Deshmukh A., Wisniewski J.R. (2015). Absolute quantitative profiling of the key metabolic pathways in slow and fast skeletal muscle. J. Proteome Res..

[42] Shevchenko A., Wilm M., Vorm O., Mann M. (1996). Mass spectrometric sequencing of proteins silver-stained polyacrylamide gels. Anal. Chem..

[43] Chen E.I., McClatchy D., Park S.K., Yates J.R. (2008). Comparisons of mass spectrometry compatible surfactants for global analysis of the mammalian brain proteome. Anal. Chem..

[44] Leon I.R., Schwammle V., Jensen O.N., Sprenger R.R. (2013). Quantitative assessment of in-solution digestion efficiency identifies optimal protocols for unbiased protein analysis. Mol. Cell. Proteom..

[45] Nagaraj N., Kulak N.A., Cox J., Neuhauser N., Mayr K., Hoerning O., Vorm O., Mann M. (2012). System-wide perturbation analysis with nearly complete coverage of the yeast proteome by single-shot ultra HPLC runs on a bench top Orbitrap. Mol. Cell. Proteom..

[46] Choudhary G., Horvath C. (1996). Ion-exchange chromatography. Methods Enzymol..

[47] Howard G.A., Martin A.J. (1950). The separation of the C12-C18 fatty acids by reversed-phase partition chromatography. Biochem. J..

[48] Hjerten S. (1981). Hydrophobic interaction chromatography of proteins, nucleic acids, viruses, and cells on noncharged amphiphilic gels. Methods Biochem. Anal..

[49] Hubner N.C., Ren S., Mann M. (2008). Peptide separation with immobilized pI strips is an attractive alternative to in-gel protein digestion for proteome analysis. Proteomics.

[50] Washburn M.P., Wolters D., Yates J.R. (2001). Large-scale analysis of the yeast proteome by multidimensional protein identification technology. Nat. Biotechnol..

[51] Rappsilber J., Mann M., Ishihama Y. (2007). Protocol for micro-purification, enrichment, pre-fractionation and storage of peptides for proteomics using StageTips. Nat. Protoc..

[52] Wisniewski J.R., Mann M. (2012). Consecutive proteolytic digestion in an enzyme reactor increases depth of proteomic and phosphoproteomic analysis. Anal. Chem..

[53] Wisniewski J.R., Prus G. (2015). Homogenous Phase Enrichment of Cysteine-Containing Peptides for Improved Proteome Coverage. Anal. Chem..

[54] Fonslow B.R., Stein B.D., Webb K.J., Xu T., Choi J., Park S.K., Yates J.R. (2013). Digestion and depletion of abundant proteins improves proteomic coverage. Nat. Methods.

[55] Fonslow B.R., Stein B.D., Webb K.J., Xu T., Choi J., Park S.K., Yates J.R. (2014). Addendum: Digestion and depletion of abundant proteins improves proteomic coverage. Nat. Methods.

[56] Kocher T., Swart R., Mechtler K. (2011). Ultra-high-pressure RPLC hyphenated to an LTQ-Orbitrap Velos reveals a linear relation between peak capacity and number of identified peptides. Anal. Chem..

[57] Hebert A.S., Richards A.L., Bailey D.J., Ulbrich A., Coughlin E.E., Westphall M.S., Coon J.J. (2014). The one hour yeast proteome. Mol. Cell. Proteom..

[58] Riley N.M., Mullen C., Weisbrod C.R., Sharma S., Senko M.W., Zabrouskov V., Westphall M.S., Syka J.E., Coon J.J. (2015). Enhanced Dissociation of Intact Proteins with High Capacity Electron Transfer Dissociation. J. Am. Soc. Mass Spectrom..

[59] Cox J., Mann M. (2008). MaxQuant enables high peptide identification rates, individualized p.p.b.-range mass accuracies and proteome-wide protein quantification. Nat. Biotechnol..

[60] Ong S.E., Mann M. (2007). Stable isotope labeling by amino acids in cell culture for quantitative proteomics. Methods Mol. Biol..

[61] Gygi S.P., Rist B., Gerber S.A., Turecek F., Gelb M.H., Aebersold R. (1999). Quantitative analysis of complex protein mixtures using isotope-coded affinity tags. Nat. Biotechnol..

[62] Boersema P.J., Aye T.T., van Veen T.A., Heck A.J., Mohammed S. (2008). Triplex protein quantification based on stable isotope labeling by peptide dimethylation applied to cell and tissue lysates. Proteomics.

[63] Eberl H.C., Spruijt C.G., Kelstrup C.D., Vermeulen M., Mann M. (2013). A map of general and specialized chromatin readers in mouse tissues generated by label-free interaction proteomics. Mol. Cell.

[64] Wisniewski J.R., Hein M.Y., Cox J., Mann M. (2014). A “proteomic ruler” for protein copy number and concentration estimation without spike-in standards. Mol. Cell. Proteom..

[65] DeFronzo R.A., Tripathy D. (2009). Skeletal muscle insulin resistance is the primary defect in Type 2 diabetes. Diabetes Care.

[66] Warram J.H., Martin B.C., Krolewski A.S., Soeldner J.S., Kahn C.R. (1990). Slow glucose removal rate and hyperinsulinemia precede the development of type II diabetes in the offspring of diabetic parents. Ann. Intern. Med..

[67] Petersen K.F., Shulman G.I. (2002). Pathogenesis of skeletal muscle insulin resistance in Type 2 diabetes mellitus. Am. J. Cardiol..

[68] Giebelstein J., Poschmann G., Hojlund K., Schechinger W., Dietrich J.W., Levin K., Beck-Nielsen H., Podwojski K., Stuhler K., Meyer H.E. (2012). The proteomic signature of insulin-resistant human skeletal muscle reveals increased glycolytic and decreased mitochondrial enzymes. Diabetologia.

[69] Zabielski P., Lanza I.R., Gopala S., Holtz Heppelmann C.J., Bergen H.R., Dasari S., Nair K.S. (2015). Altered skeletal muscle mitochondrial proteome as the basis of disruption of mitochondrial function in diabetic mice. Diabetes.

[70] Stentz F.B., Kitabchi A.E. (2007). Transcriptome and proteome expressions involved in insulin resistance in muscle and activated T-lymphocytes of patients with type 2 diabetes. Genom. Proteom. Bioinform..

[71] Mullen E., O’Reilly E., Ohlendieck K. (2011). Skeletal muscle tissue from the Goto-Kakizaki rat model of Type-2 diabetes exhibits increased levels of the small heat shock protein Hsp27. Mol. Med. Rep..

[72] Hwang H., Bowen B.P., Lefort N., Flynn C.R., De Filippis E.A., Roberts C., Smoke C.C., Meyer C., Hojlund K., Yi Z., Mandarino L.J. (2010). Proteomics analysis of human skeletal muscle reveals novel abnormalities in obesity and Type 2 diabetes. Diabetes.

[73] Choi M., Choi J.W., Chaudhari H.N., Aseer K.R., Mukherjee R., Yun J.W. (2013). Gender-dimorphic regulation of skeletal muscle proteins in streptozotocin-induced diabetic rats. Cell. Physiol. Biochem..

[74] Al-Khalili L., de Castro Barbosa T., Ostling J., Massart J., Cuesta P.G., Osler M.E., Katayama M., Nystrom A.C., Oscarsson J., Zierath J.R. (2014). Proteasome inhibition in skeletal muscle cells unmasks metabolic derangements in Type 2 diabetes. Am. J. Physiol. Cell Physiol..

[75] Hojlund K., Bowen B.P., Hwang H., Flynn C.R., Madireddy L., Geetha T., Langlais P., Meyer C., Mandarino L.J., Yi Z. (2009). *In vivo* phosphoproteome of human skeletal muscle revealed by phosphopeptide enrichment and HPLC-ESI-MS/MS. J. Proteome Res..

[76] Zhao X., Leon I.R., Bak S., Mogensen M., Wrzesinski K., Hojlund K., Jensen O.N. (2011). Phosphoproteome analysis of functional mitochondria isolated from resting human muscle reveals extensive phosphorylation of inner membrane protein complexes and enzymes. Mol. Cell. Proteom..

[77] Humphrey S.J., Azimifar S.B., Mann M. (2015). High-throughput phosphoproteomics reveals *in vivo* insulin signaling dynamics. Nat. Biotechnol..

[78] Lundby A., Lage K., Weinert B.T., Bekker-Jensen D.B., Secher A., Skovgaard T., Kelstrup C.D., Dmytriyev A., Choudhary C., Lundby C. (2012). Proteomic analysis of lysine acetylation sites in rat tissues reveals organ specificity and subcellular patterns. Cell Rep..

[79] Lundby A., Secher A., Lage K., Nordsborg N.B., Dmytriyev A., Lundby C., Olsen J.V. (2012). Quantitative maps of protein phosphorylation sites across 14 different rat organs and tissues. Nat. Commun..

[80] Zhang X., Ma D., Caruso M., Lewis M., Qi Y., Yi Z. (2014). Quantitative phosphoproteomics reveals novel phosphorylation events in insulin signaling regulated by protein phosphatase 1 regulatory subunit 12A. J. Proteom..

[81] Parker C.E., Mocanu V., Mocanu M., Dicheva N., Warren M.R., Alzate O. (2010). Mass Spectrometry for Post-Translational Modifications. Neuroproteomics.

[82] Karpe F., Dickmann J.R., Frayn K.N. (2011). Fatty acids, obesity, and insulin resistance: Time for a reevaluation. Diabetes.

[83] Dresner A., Laurent D., Marcucci M., Griffin M.E., Dufour S., Cline G.W., Slezak L.A., Andersen D.K., Hundal R.S., Rothman D.L. (1999). Effects of free fatty acids on glucose transport and IRS-1-associated phosphatidylinositol 3-kinase activity. J. Clin. Investig..

[84] Hawley J.A. (2004). Exercise as a therapeutic intervention for the prevention and treatment of insulin resistance. Diabetes Metab. Res. Rev..

[85] Petersen K.F., Dufour S., Befroy D., Garcia R., Shulman G.I. (2004). Impaired mitochondrial activity in the insulin-resistant offspring of patients with type 2 diabetes. N. Engl. J. Med..

[86] Schrauwen P., Hesselink M.K. (2004). Oxidative capacity, lipotoxicity, and mitochondrial damage in Type 2 diabetes. Diabetes.

[87] Oberbach A., Bossenz Y., Lehmann S., Niebauer J., Adams V., Paschke R., Schon M.R., Bluher M., Punkt K. (2006). Altered fiber distribution and fiber-specific glycolytic and oxidative enzyme activity in skeletal muscle of patients with Type 2 diabetes. Diabetes Care.

[88] Freemont A.J., Hoyland J.A. (2007). Morphology, mechanisms and pathology of musculoskeletal ageing. J. Pathol..

[89] Leenders M., Verdijk L.B., van der Hoeven L., Adam J.J., van Kranenburg J., Nilwik R., van Loon L.J. (2013). Patients with Type 2 diabetes show a greater decline in muscle mass, muscle strength, and functional capacity with aging. J. Am. Med. Dir Assoc..

[90] Andersen H., Nielsen S., Mogensen C.E., Jakobsen J. (2004). Muscle strength in Type 2 diabetes. Diabetes.

[91] Park S.W., Goodpaster B.H., Strotmeyer E.S., de Rekeneire N., Harris T.B., Schwartz A.V., Tylavsky F.A., Newman A.B. (2006). Decreased muscle strength and quality in older adults with type 2 diabetes: The health, aging, and body composition study. Diabetes.

[92] Harris M.I. (1993). Undiagnosed NIDDM: Clinical and public health issues. Diabetes Care.

[93] Herman W.H., Ye W., Griffin S.J., Simmons R.K., Davies M.J., Khunti K., Rutten G.E., Sandbaek A., Lauritzen T., Borch-Johnsen K. (2015). Early Detection and Treatment of Type 2 Diabetes Reduce Cardiovascular Morbidity and Mortality: A Simulation of the Results of the Anglo-Danish-Dutch Study of Intensive Treatment in People With Screen-Detected Diabetes in Primary Care (ADDITION-Europe). Diabetes Care.

[94] Zendjabil M. (2015). Biological diagnosis of diabetes mellitus. Pathol. Biol..

[95] Stumpf M.P., Thorne T., de Silva E., Stewart R., An H.J., Lappe M., Wiuf C. (2008). Estimating the size of the human interactome. Proc. Natl. Acad. Sci. USA.

[96] Hein M.Y., Hubner N.C., Poser I., Cox J., Nagaraj N., Toyoda Y., Gak I.A., Weisswange I., Mansfeld J., Buchholz F. (2015). A Human Interactome in Three Quantitative Dimensions Organized by Stoichiometries and Abundances. Cell.

[97] Mellacheruvu D., Wright Z., Couzens A.L., Lambert J.P., St-Denis N.A., Li T., Miteva Y.V., Hauri S., Sardiu M.E., Low T.Y. (2013). The CRAPome: A contaminant repository for affinity purification-mass spectrometry data. Nat. Methods.

[98] Keilhauer E.C., Hein M.Y., Mann M. (2015). Accurate protein complex retrieval by affinity enrichment mass spectrometry (AE-MS) rather than affinity purification mass spectrometry (AP-MS). Mol. Cell. Proteom..

[99] Nesvizhskii A.I. (2012). Computational and informatics strategies for identification of specific protein interaction partners in affinity purification mass spectrometry experiments. Proteomics.

[100] Caruso M., Ma D., Msallaty Z., Lewis M., Seyoum B., Al-janabi W., Diamond M., Abou-Samra A.B., Hojlund K., Tagett R. (2014). Increased interaction with insulin receptor substrate 1, a novel abnormality in insulin resistance and Type 2 diabetes. Diabetes.

[101] Krogh-Madsen R., Thyfault J.P., Broholm C., Mortensen O.H., Olsen R.H., Mounier R., Plomgaard P., van Hall G., Booth F.W., Pedersen B.K. (2010). A 2-wk reduction of ambulatory activity attenuates peripheral insulin sensitivity. J. Appl. Physiol. (1985).

[102] Olsen R.H., Krogh-Madsen R., Thomsen C., Booth F.W., Pedersen B.K. (2008). Metabolic responses to reduced daily steps in healthy nonexercising men. JAMA.

[103] Garetto L.P., Richter E.A., Goodman M.N., Ruderman N.B. (1984). Enhanced muscle glucose metabolism after exercise in the rat: The two phases. Am. J. Physiol..

[104] Richter E.A., Garetto L.P., Goodman M.N., Ruderman N.B. (1984). Enhanced muscle glucose metabolism after exercise: Modulation by local factors. Am. J. Physiol..

[105] Holloszy J.O. (2005). Exercise-induced increase in muscle insulin sensitivity. J. Appl. Physiol. (1985).

[106] Christ-Roberts C.Y., Pratipanawatr T., Pratipanawatr W., Berria R., Belfort R., Mandarino L.J. (2003). Increased insulin receptor signaling and glycogen synthase activity contribute to the synergistic effect of exercise on insulin action. J. Appl. Physiol. (1985).

[107] Wallberg-Henriksson H., Holloszy J.O. (1984). Contractile activity increases glucose uptake by muscle in severely diabetic rats. J. Appl. Physiol. Respir. Environ. Exerc. Physiol..

[108] Deshmukh A.S., Hawley J.A., Zierath J.R. (2008). Exercise-induced phospho-proteins in skeletal muscle. Int. J. Obes..

[109] Hoffman N.J., Parker B.L., Chaudhuri R., Fisher-Wellman K.H., Kleinert M., Humphrey S.J., Yang P., Holliday M., Trefely S., Fazakerley D.J. (2015). Global Phosphoproteomic Analysis of Human Skeletal Muscle Reveals a Network of Exercise-Regulated Kinases and AMPK Substrates. Cell Metab..

[110] McGee S.L., Fairlie E., Garnham A.P., Hargreaves M. (2009). Exercise-induced histone modifications in human skeletal muscle. J. Physiol..

[111] Knowler W.C., Barrett-Connor E., Fowler S.E., Hamman R.F., Lachin J.M., Walker E.A., Nathan D.M. (2002). Diabetes Prevention Program Research G. Reduction in the incidence of Type 2 diabetes with lifestyle intervention or metformin. N. Engl. J. Med..

[112] Coffey V.G., Hawley J.A. (2007). The molecular bases of training adaptation. Sports Med..

[113] Adams G.R., Hather B.M., Baldwin K.M., Dudley G.A. (1993). Skeletal muscle myosin heavy chain composition and resistance training. J. Appl. Physiol..

[114] Widrick J.J., Stelzer J.E., Shoepe T.C., Garner D.P. (2002). Functional properties of human muscle fibers after short-term resistance exercise training. Am. J. Physiol. Regul. Integr. Comp. Physiol..

[115] Spina R.J., Chi M.M., Hopkins M.G., Nemeth P.M., Lowry O.H., Holloszy J.O. (1996). Mitochondrial enzymes increase in muscle in response to 7–10 days of cycle exercise. J. Appl. Physiol. (1985).

[116] Green H.J., Helyar R., Ball-Burnett M., Kowalchuk N., Symon S., Farrance B. (1992). Metabolic adaptations to training precede changes in muscle mitochondrial capacity. J. Appl. Physiol. (1985).

[117] Benziane B., Burton T.J., Scanlan B., Galuska D., Canny B.J., Chibalin A.V., Zierath J.R., Stepto N.K. (2008). Divergent cell signaling after short-term intensified endurance training in human skeletal muscle. Am. J. Physiol. Endocrinol. Metab..

[118] Pilegaard H., Saltin B., Neufer P.D. (2003). Exercise induces transient transcriptional activation of the PGC-1alpha gene in human skeletal muscle. J. Physiol..

[119] Holloway K.V., O’Gorman M., Woods P., Morton J.P., Evans L., Cable N.T., Goldspink D.F., Burniston J.G. (2009). Proteomic investigation of changes in human vastus lateralis muscle in response to interval-exercise training. Proteomics.

[120] Egan B., Dowling P., O’Connor P.L., Henry M., Meleady P., Zierath J.R., O’Gorman D.J. (2011). 2-D DIGE analysis of the mitochondrial proteome from human skeletal muscle reveals time course-dependent remodelling in response to 14 consecutive days of endurance exercise training. Proteomics.

[121] Schild M., Ruhs A., Beiter T., Zugel M., Hudemann J., Reimer A., Krumholz-Wagner I., Wagner C., Keller J., Eder K. (2015). Basal and exercise induced label-free quantitative protein profiling of m. vastus lateralis in trained and untrained individuals. J. Proteom..

[122] Alves R.M., Vitorino R., Figueiredo P., Duarte J.A., Ferreira R., Amado F. (2010). Lifelong physical activity modulation of the skeletal muscle mitochondrial proteome in mice. J. Gerontol. A Biol. Sci. Med. Sci..

[123] Moriggi M., Vasso M., Fania C., Capitanio D., Bonifacio G., Salanova M., Blottner D., Rittweger J., Felsenberg D., Cerretelli P. (2010). Long term bed rest with and without vibration exercise countermeasures: Effects on human muscle protein dysregulation. Proteomics.

[124] Hussey S.E., Sharoff C.G., Garnham A., Yi Z., Bowen B.P., Mandarino L.J., Hargreaves M. (2013). Effect of exercise on the skeletal muscle proteome in patients with Type 2 diabetes. Med. Sci. Sports Exerc..

[125] Yuan H., Niu Y., Liu X., Yang F., Niu W., Fu L. (2013). Proteomic analysis of skeletal muscle in insulin-resistant mice: Response to 6-week aerobic exercise. PLoS ONE.

[126] Pedersen B.K., Febbraio M.A. (2012). Muscles, exercise and obesity: Skeletal muscle as a secretory organ. Nat. Rev. Endocrinol..

[127] Meissner F., Scheltema R.A., Mollenkopf H.J., Mann M. (2013). Direct proteomic quantification of the secretome of activated immune cells. Science.

[128] Deshmukh A.S., Cox J., Jensen L.J., Meissner F., Mann M. (2015). Secretome Analysis of Lipid-Induced Insulin Resistance in Skeletal Muscle Cells by a Combined Experimental and Bioinformatics Workflow. J. Proteome Res..

[129] Deshmukh A., Salehzadeh F., Metayer-Coustard S., Fahlman R., Nair K.S., Al-Khalili L. (2009). Post-transcriptional gene silencing of ribosomal protein S6 kinase 1 restores insulin action in leucine-treated skeletal muscle. Cell. Mol. Life Sci..

[130] Dimopoulos N., Watson M., Sakamoto K., Hundal H.S. (2006). Differential effects of palmitate and palmitoleate on insulin action and glucose utilization in rat L6 skeletal muscle cells. Biochem. J..

[131] Huang C., Somwar R., Patel N., Niu W., Torok D., Klip A. (2002). Sustained exposure of L6 myotubes to high glucose and insulin decreases insulin-stimulated GLUT4 translocation but upregulates GLUT4 activity. Diabetes.

[132] Steinberg G.R., Michell B.J., van Denderen B.J., Watt M.J., Carey A.L., Fam B.C., Andrikopoulos S., Proietto J., Gorgun C.Z., Carling D. (2006). Tumor necrosis factor alpha-induced skeletal muscle insulin resistance involves suppression of AMP-kinase signaling. Cell Metab..

[133] Nedachi T., Fujita H., Kanzaki M. (2008). Contractile C2C12 myotube model for studying exercise-inducible responses in skeletal muscle. Am. J. Physiol. Endocrinol. Metab..

[134] Raschke S., Eckardt K., Bjorklund Holven K., Jensen J., Eckel J. (2013). Identification and validation of novel contraction-regulated myokines released from primary human skeletal muscle cells. PLoS ONE.

